# Extranodal nasal-type natural killer/T-cell lymphoma with penile involvement: a case report and review of the literature

**DOI:** 10.1186/s12894-017-0273-8

**Published:** 2017-09-06

**Authors:** Xiaotian Wang, Zimu Gong, Shawn Xiang Li, Wei Yan, Yongsheng Song

**Affiliations:** 1 Department of Urology, Shengjing Hospital of China Medical University, 36 Saohao Road, Shenyang, Liaoning China; 2 Department of Hematology, Shengjing Hospital of China Medical University, Shenyang, China; 3 International College, China Medical University, Shenyang, China

**Keywords:** NK/T lymphoma, Penile malignancy

## Abstract

**Background:**

Extranodal natural killer/T-cell lymphoma (ENKTL) usually presents as a localized disease in the nasal cavity; extension to the male genitourinary system is very rare and has been characterized only recently. Most cases present with predominantly extranodal involvement, advanced stage disease, highly aggressive course, and strong association with Epstein-Barr virus (EBV). While metastasis is common in ENKTLs, the penis is rarely involved in both nasal and non-nasal ENKTLs and only one report was published to date.

**Case presentation:**

One patient with NK/T-cell lymphoma, presented initially with a penile mass, is reported. The 58-year-old man who presented with progressive painless penile swelling underwent penectomy for penile tumor. Histologically, the glans and foreskin revealed neoplastic infiltration of medium-sized lymphoma cells expressing CD56, CD3, granzyme-B, and labeled for EBV-encoded RNA in situ hybridization. Findings were consistent with NK/T-cell lymphoma. By detailed history, we learned that the patient had nasal obstruction for more than 10 years. Nasopharyngeal involvement was screened with PET-CT; ENKTL was diagnosed after a nasopharyngeal biopsy. The final diagnosis was primary nasal NK/T-cell lymphoma, with metastasis to the penis. Additional sites of disease appeared soon afterward (adrenal gland, liver, spleen and lymph nodes). The patient died within 4 months.

**Conclusion:**

This study suggested that penile NK/T-cell lymphoma tends to disseminate early and pursues an aggressive course. It is imperative to distinguish nasal NK/T lymphoma from other types of tumors, because the prognosis and treatment differ significantly for secondary metastases.

## Background

Extranodal natural killer/T-cell lymphoma (ENKTL) is a distinct entity of non-Hodgkin lymphoma with unique characteristics in both biological features and clinical behavior. ENKTL is most commonly seen in young to middle-aged patients, with a male preponderance of 2–4:1. Despite its rarity in the Caucasian population, ENKTL had a significantly higher incidence in East Asia and Latin America [1].

The common and primary sites which ENKTL arises from include the nasal cavity, upper aerodigestive tract, and other midline facial structures [2]. Invasion to adjacent tissues and metastasis to distant organs are also common [3, 4]. Overall, ENKTL is associated with an aggressive disease course and poor clinical outcome, especially with metastatic disease beyond the nasal cavity, in which the complete remission rate is only about 30%, and median survival is 4.3 months [5].

Since the primary lesion is often occult without causing significant symptoms, ENKTL is often diagnosed in relatively advanced stages when metastatic lesions occur. The most commonly involved metastatic sites include skin, gastrointestinal tract, testis, kidney, and breast. The morphological characteristics of metastatic lesions are often less typical than that of the primary lesion, and extensive necrosis further compromises the accuracy of histopathological diagnosis. Therefore, it is crucial to investigate potential nasal lesion when metastatic ENKTL is suspected.

We herein report a case of penile metastatic NK/T cell lymphoma in which the diagnosis was established first at the metastatic site, and the primary lesion in the nasal cavity was uncovered later.

## Case presentation

### Clinical history

A 58-year-old male was admitted to our hospital with diffuse swelling of the penis, followed by sclerosis and ischemic changes in the glans and anterior segment of the penis, without bloody or purulent discharge. On review of systems, he denied fever, night sweat, and weight loss. Past medical and family history are not contributory. On admission, his vital signs were within normal limits. On physical examination, his face was thin and worn. No lymphadenopathy was palpated in the neck and supraclavicular region; visual examination of the genitals manifested a diffused enlargement of the penis with local ulcer and necrotic tissue in the glans penis.

Laboratory findings showed the white blood cell count as 8100/mm^3^, hemoglobin as 12.5 g/dL, and a platelet count of 276,000/mm^3^, and liver function, kidney function, and the serum lactate dehydrogenase (LDH) levels were within normal limits. The EBV IgG was positive in the serum, the EBV DNA determination was negative in the blood, and CRP was 22.9 mg/mL.

**Fig. 1:**
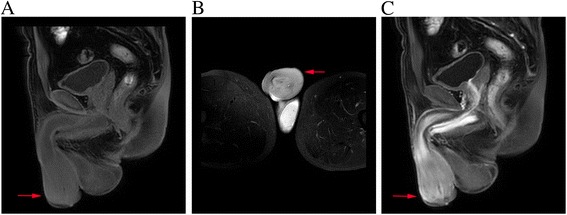
**a** Sagittal T1-weighted magnetic resonance imaging scan showing mass around the corpus cavernosum (arrow); **b** Axial T2-weight magnetic resonance imaging scan showing ill-defined mass around the corpus cavernosum (arrow); **c** Sagittal T1-weighted fat suppression contrast-enhanced magnetic resonance imaging scan showing enhanced mass around the corpus cavernosum (arrow)

Pelvic enhanced MRI showed space-occupying lesions in the forward part of the penis, urethral orifice stenosis, and cavernosal swelling (Fig. 1a-c). No lesion in the bladder was identified upon cystoscopy. CT scans of chest and abdomen did not detect enlarged lymph nodes. The liver and spleen were of normal size and shape. Bone marrow biopsy showed lymphoma cell infiltration up to 3.2%.

### Pathologic finding and treatment

**Fig. 2:**
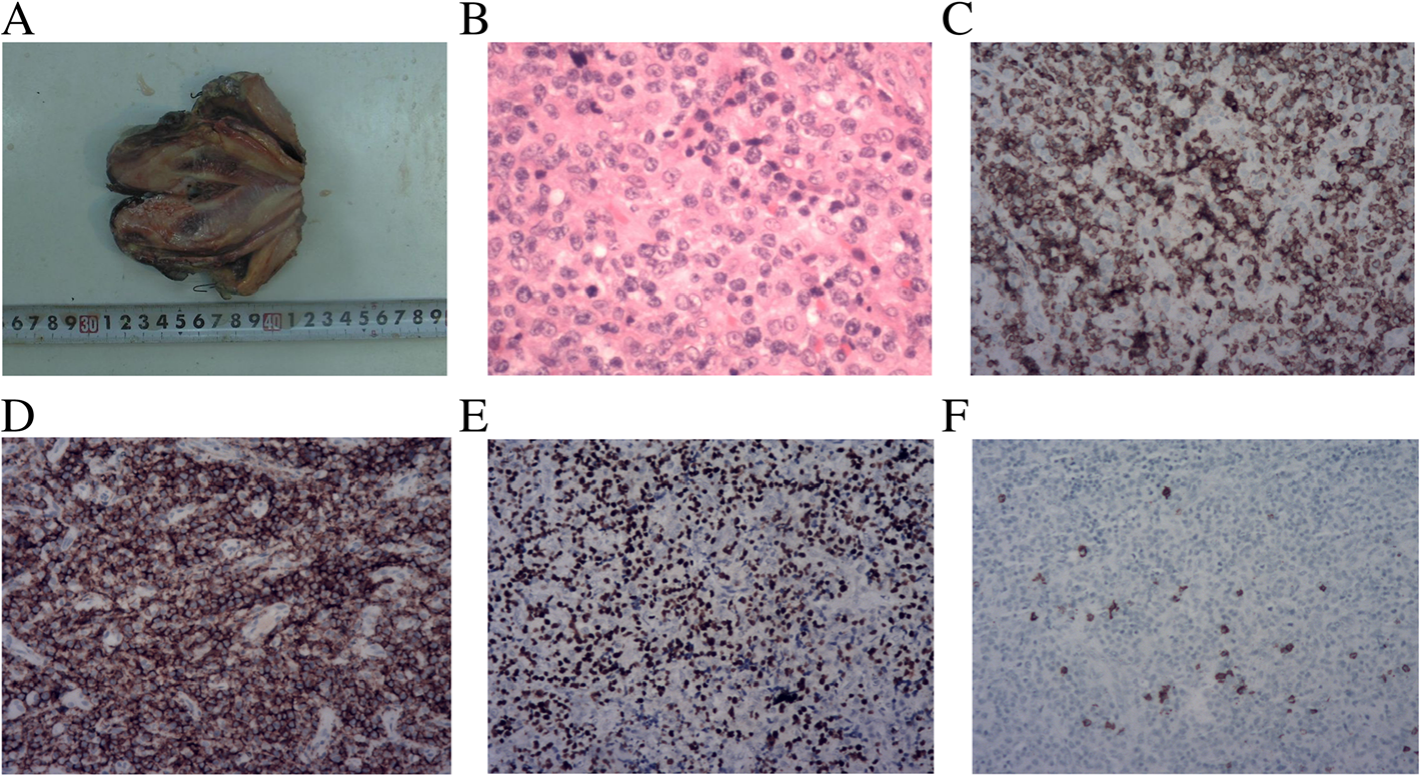
Pathologic gross (**a**) and microscopic features of thespecimen. **b** Histological examinations stained with hematoxylin and eosin revealed infiltration of medium-sized cells with irregular nuclei, inconspicuous nucleoli, and many apoptotic bodies(H&E stains × 100). **c** Immunohistochemical staining showed positive reactivity for CD3(×100). **d** Immunohistochemical staining showed positive CD56(×100). **e** In situ hybridization for Epstein-Barr virus-encoded RNA showed positive reaction in atypical cells(×100). **f** Immunohistochemical staining showed negative CD20(×100)

Microscopic findings of the penile mass showed infiltration of medium-sized cells with irregular nuclei, inconspicuous nucleoli, and many apoptotic bodies. The lymphoma cells exhibited a notable angioinfiltrative growth pattern, with homocentric arrangement around small arteries, and coagulative necrosis was also observed (Fig. 2a and b). On immunohistochemical staining, the atypical cells were positive for CD3, CD56, vimentin, LCA, TIA-1, and granzyme B, but negative for cytokeratin, CD20, bcl-2, bcl-6, and ALK (Fig. 2c-e). In the case of in situ hybridization for EBV encoded RNA (EBER), most atypical cells were also labeled (Fig. 2f). These findings were consistent with NK/T-cell lymphoma.

**Fig. 3:**
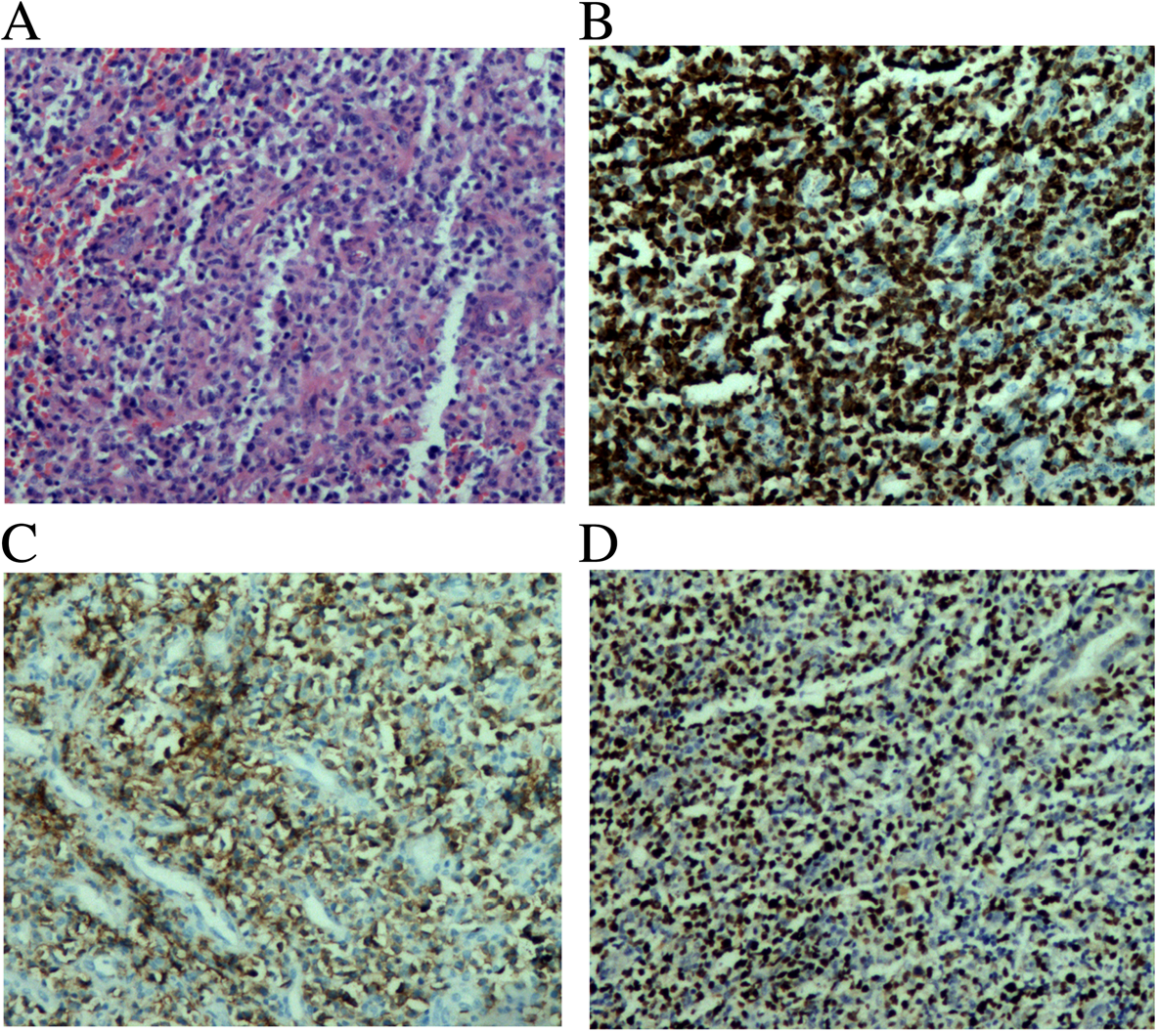
**a** Pathological features of sinoscopical biopsy showed diffuse infiltration of small to medium-sized lymphoid cells with angiodestructive growth pattern, and apoptotic bodies were present (H&E stains ×100). **b** Immunohistochemical staining showed positive reactivity for CD3(×100). **c** Immunohistochemical staining showed positive CD56(×100). **d** In situ hybridization for Epstein-Barr virus-encoded RNA showed positive reaction in atypical cells(×100)

By detailed history, we learned that the patient had nasal obstruction for more than 10 years. PET-CT found a high-FDG metabolic mass in the nasal cavity and the nasopharyngeal cavity. Space-occupying lesion in the nasal cavity was observed and biopsied with sinoscopy. Histological examination showed diffuse infiltration of small to medium-sized lymphoid cells with angio-destructive growth pattern, and apoptotic bodies were present (Fig. 3a). The tumor cells were positive for CD3, CD56, EBER; they were negative for CD20 and granzyme B (Fig. 3. b-d). Based on the endoscopic and immunophenotypical findings, the diagnosis of penile metastasis from extranodal nasal-type NK/T-cell lymphoma was established.

Twenty days after neoplasm resection, the patient consented to chemotherapy treatment followed by radiotherapy and autologous stem cell transplantation. CHOP-L regimen (cyclophosphamide, doxorubicin, vincristine, prednisone, and L-asparaginase) was planned to be given every 3 weeks, with six cycles in total. However, the third cycle of chemotherapy was delayed due to ascites, fever, and grade 4 myelosuppression. Subsequently, he developed tachypnea, abdominal pain, fever, and diarrhea. According to the CT results, His lymphoma was refractory to chemotherapy and metastases developed in the adrenal gland, liver, spleen and lymph nodes. The patient died of disease 1 month later, which is approximately 4 months after the diagnosis was made.

Informed consent was obtained from the patient for publication of this case report and any accompanying image.

## Discussion

Lymphoma of the NK cell phenotype is rare. International T-cell Lymphoma Project reported that 10.4% of T cell lymphoma was of the NK/T-cell type [6]. WHO recognized entities include extranodal NK/T-cell lymphoma, nasal type, aggressive NK cell leukemia, and chronic lymphoproliferative disorders of NK cells [7]. Among these entities, the last one is indolent but the other two exhibit an aggressive course and short survival. Diagnosis of NK cell lymphoma may be challenging. The characteristic immunophenotype of NK/T-cell lymphoma is CD56+, CD2+, surface CD3-, LCA+. Also, CD56 is a useful marker [8]. EBER is a diagnostic requisite of NK/T-cell lymphoma and is especially useful in metastatic sites where the CD56 expression might be lost [9].

NK/T-cell lymphoma often occurs in the nasal cavity and the upper aerodigestive tract, and it is associated with a poor prognosis. Bone marrow involvement occurs in <10% of patients. Distant metastasis of NK/T-cell lymphoma is sporadically reported, with the most commonly involved site including skin, testis, gastrointestinal tract, eyes, lungs, adrenal glands, breast, and brain [10, 11]. ENKTL with penis involvement is very rare, with only one case reported. We reviewed these ENKTL cases with involvement of male genitourinary system, whose clinical characteristics are summarized in Table 1 [12–27].

**Table 1:** Clinical features of ENKTL cases with involvement of male genitourinaty system

Location	Age	Presentation	Treatment	Outcome	Ref
Kidney	72	Left flank pain	RT	DOD	[12]
Kidney	35	Urinary symptoms	CT	DOD	[13]
Prostate	59	Fever and dysuria	CT	DOD	[14]
Testis	38	Nasal granuloma	Orchiectomy RT,CT	DOD	[15]
Testis	61	Right testicular swelling	Orchiectomy, CT	AWR	[16]
Testis	68	Left testicular swelling	Orchiectomy, CT	NA	[16]
Testis	40	Left testicular swelling with B symptom	Orchiectomy, CT	DOD	[16]
Testis	44	Bilateral testicular swelling with B symptom	Orchiectomy, CT	DOD	[16]
Testis	45	Left testicular swelling	Orchiectomy, CT	NA	[16]
Testis	13	Right testicular swelling with B symptom	Orchiectomy, CT	NA	[16]
Testis	22	Right testicular swelling with B symptom	Orchiectomy, CT	AWD	[16]
Testis	0.67	Enlarged scrotum	CT, CBSCT	AWD	[17]
Testis	28	Painful testicular mass	CT	DOD	[18]
Testis	76	Left painless testicular enlargement	Orchiectomy, CT	AWD	[19]
Testis	36	Left testicular swelling fatigue weight-loss night sweat	CT, RT, allo-SCT	AWR	[20]
Testis	81	Right thigh soft tissue mass	Bilateral orchiectomy	DOD	[21]
Testis	30	Bilateral scrotal swelling and fever	RT, CT	DOD	[22]
Testis	52	Right testicle swelling	Chemo/RT	DOD	[23]
Testis	66	Painless enlargement of right testis	Orchiectomy	AWD	[24]
Testis	35	Right testicular swelling	Orchiectomy, CT	NA	[25]
Testis	47	Left scrotal swelling, weight loss	RT, CT	DOD	[26]
Testis	71	Nontender left testicular mass	Orchiectomy	DOD	[26]
Testis	55	Left scrotal swelling, fever and weight loss	Orchiectomy and CT	DOD	[26]
Penis	48	A painful mass in the penile shaft	CT	AWR	[27]

Abbreviations: *ENKTL* extranodal natural killer/T-cell lymphoma, *CT* chemotherapy, *RT* radiotherapy, *allo-SCT* allogeneic stem cell transplantation, *CBSCT* cord blood stem cell transplantation, *AWR* alive with relapse, *DOD* died of disease, *AWD* alive with disease, *NA* not available

Lymphoma of the penis can present as a solitary nodule, non-healing ulcer, or diffuse penile swelling [28]. Upon reviewing the literature, most reported cases of penile lymphoma were classified as diffuse large B-cell lymphoma (DLBCL) [29]. Primary penile NK/T-cell lymphoma is never reported. In combination with clinical history and pathological results, the final diagnosis of penile metastasis secondary to extranodal nasal-type NK/T-cell lymphoma was established in our case. The diagnosis of the rare diseases has always been a challenging process. The final diagnosis of lymphoma of the penis was made on the histopathological examination of tissue biopsy. Histological analyses must include comprehensive immunohistochemistry tests to differentiate lymphoma from undifferentiated sarcomas or carcinoma and to distinguish between different types of lymphomas.

Nasal type NK/T cell lymphoma is associated with poor prognosis and often follows a rapidly progressive course. Age, symptoms, local tumor invasion, clinical stage, treatment response, NK/T cell IPI, LDH, and EBV infection were reported to be prognostic in ENKTL [30]. Although lacking statistical data, some literature reported distant metastasis and bone marrow involvement, with a very poor prognosis [31]. Our patient had unfavorable prognostic factors (EBV infection, distant metastasis, and bone marrow involvement), and the available data on optimal treatment strategies are limited. Systemic chemotherapy and involved field radiotherapy is the current standard of care for nasal-type NK/T-cell lymphoma, and CHOP is a very commonly used regimen.

The introduction of L-asparaginase-containing regimens led to further improvement in outcome, as most studies using L-asparaginase-containing regimens in a relapsed or refractory setting reported response rates of around 50% [32]. Therefore, the use of L-asparaginase-containing regimens may be superior to the use of CHOP regimen alone. The prognosis of ENKTL involving male genitourinary system is poor: most reported that patients died due to complications of the disease or of the treatment. Patients need chemotherapy combined with other effective treatments. A recent study showed that autologous stem cell transplantation is a feasible and effective therapy for high-risk nasal-type NK/T-cell lymphoma. We reviewed these trials, whose clinical outcomes are summarized in Table 2 [33–38]. The patient was also recommended to undergo autologous stem cell transplantation. However, unfortunately, he rapidly died due to disease progression, before that could be possible.

**Table 2:** Clinical outcomes of autologous stem cell transplantation in patients with ENKTL

Author	Cases	Median age	Male:female	Median follow-up(M)	Outcome	Ref
Fox	28	47	17:11	33	1-year NRM:11%,2-year PFS:41%	[33]
Yhim	62	45.5	43:19	43.3	CR:61.3%; PR:38.7% 3-year PFS:52.4%,3-year OS:60%	[34]
Wang	31	43	22:9	70	ORR:90.3%,1-year ORR:96.8%	[35]
Lee	47	42	34:13	116.5	5-year OS:56% In people who were in CR: disease-specific 5-year OS:87.3%	[36]
Cui	22	38	38:19	24	5-year OS:79.3% 5-year-PFS:36.4%	[37]
Kim	16	36	5:3	22.4	2-year OS:71.3%,RFS:25.8%	[38]

Abbreviations: *NRM* non-relapse mortality, *PFS* progression-free survival, *ORR* overall response rate, *OS* overall survival, *CR* complete response, *PR* partial response, *RFS* relapse free survival

There are certain strengths and a major limitation to this study. An obvious strength is that this is a case report of a very rare case that can illuminate future treatment of similar cases. The treatment plans may be of value to future clinical guidelines. The primary limitation is self-evident. This is a case report of a single case; single-case reports are inherently limited. Our treatment regimen did not work effectively. With the hindsight of this case, more effective treatments may be developed in the future.

## Conclusion

In conclusion, we present a case with primary NK/T-cell lymphoma with metastasis to the penis. The incidence of such condition is extremely low but has significant clinical value. This case broadened the spectrum of differential diagnosis of penile tumors and exhibited the value of detailed history taking and comprehensive workup. After all, patients with suspected metastasized ENKTL should undergo appropriate workup to investigate possible nasal lesions, and early biopsy should be done for suspected lesions to avoid delay in diagnosis. Due to the rapid progress and poor prognosis of this malignant disease, doctors need to make joint efforts to develop more effective therapeutic regimens.

## Abbreviations

CHOP-L: Cyclophosphamide, doxorubicin, vincristine, prednisone, L-asparaginase
DLBCL: Diffuse large B-cell lymphoma
EBER: EBV encoded RNA
EBV: Epstein-Barr virus
ENKTL: Extranodal NK/T-cell lymphoma
LDH: Lactose dehydrogenase

## References

[1] Gill H, Liang RH, Tse E (2010). Extranodal natural-killer/T-cell lymphoma, nasal type. Adv Hematol.

[2] Kwong YL (2011). The diagnosis and management of extranodal NK/T-cell lymphoma, nasal-type and aggressive NK-cell leukemia. J Clin Exp Hematop.

[3] Lim ST, Hee SW, Quek R (2008). Comparative analysis of extranodal NK/T-cell lymphoma and peripheral T-cell lymphoma: significant differences in clinical characteristics and prognosis. Eur J Haematol.

[4] Li S, Feng X, Li T (2013). Extranodal NK/T-cell lymphoma, nasal type: a report of 73 cases at MD Anderson Cancer Center. Am J Surg Pathol.

[5] Au WY, Weisenburger DD, Intragumtomchai T (2009). Clinical differences between nasal and extranasal natural killer/T-cell lymphoma: a study of 136 cases from the international peripheral T-cell lymphoma project. Blood.

[6] Savage NM, Johnson RC, Natkunam Y (2013). The spectrum of lymphoblastic, nodal and extranodal T-cell lymphomas: characteristic features and diagnostic dilemmas. Hum Pathol.

[7] Chan JKC, Quintanilla-Martinez L, Ferry JA, Swerdlow SH, Campo E, Harris NK (2008). Extranodal NK/T-cell lymphoma, nasal type. World Health Organization classification of Tumours of Haematopoietic and lymphoid tissues.

[8] Chan JK, Sin VC, Wong KF (1997). Nonnasal lymphoma expressing the natural killer cell marker CD56: a clinicopathologic study of 49 cases of an uncommon aggressive neoplasm. Blood.

[9] Au WY, Pang A, Choy C (2004). Quantification of circulating Epstein-Barr virus(EBV) DNA in the diagnosis and monitoring of natural killer cell and EBV-positive lymphomas in immunocompetent patients. Blood.

[10] Baykal C, Polat Ekinci A, Öztürk Sarı S (2016). Annular erythematous patches as the presenting sign of extranodal natural killer/T-cell lymphoma. Yurk J Hematol.

[11] Tse E, Kwong YL (2015). Nasal NK/T-cell lymphoma: RT, CT, or both. Blood.

[12] Sem Liew M, Chan AM, Galloway S (2010). Extra-nasal NK/T cell lymphoma masquerading as renal infarction. Leuk Lymphoma.

[13] Thompson MA, Habra MA, Routbort MJ (2007). Primary adrenal natural killer/T-cell nasal type lymphoma: first case report in adults. Am J Hematol.

[14] Jiang Q, Liu S, Peng J (2013). An extraordinary T/NK lymphoma, nasal type, occurring primarily in the prostate gland with unusual CD30 positivity case report and review of the literature. Diagn Pathol.

[15] Naboush A, Farhat F, Nasser SM (2013). Bifocal presentation of primary testicular extranodal NK/T-cell lymphoma: a case report and review of the literature. Case Rep Oncol Med.

[16] Liang DN, Yang ZR, Wang WY (2012). Extranodal nasal type natural killer/T-cell lymphoma of testis: report of seven cases with review of literature. Leuk Lymphoma.

[17] Yagasaki H, Ohashi H, Ito M (2011). A novel mechanism of transplacental cancer transmission: natural killer/T-cell lymphoma in the paratesticular region is of maternal origin. Blood.

[18] Ayadi L, Makni S, Toumi N (2010). Aggressive nasal-type natural killer/T-cell lymphoma associated with Ebstein Barr virus presenting as testicular tumor. Tunis Med.

[19] Mastuda M, Iwanaga T, Hashimoto S (2009). Primary Epstein-Barr virus-negative nasal-type natural killer/T-cell lymphoma of the testis. Leuk Res.

[20] Ornstein DL, Bifulco CB, Braddock DT (2008). Histopathologic and molecular aspects of CD56+ natural killer/T-cell lymphoma of the testis. Int J Surg Pathol.

[21] Morelli L, Pisctoli I, Licci S (2007). T/natural killer cell lymphoma of the testis with cutaneous and subcutaneous doft tissue involvement: a management problem. Ann Hematol.

[22] Ballereau C, Leroy X, Morschhauser F (2005). Testicular natural killer T-cell lymphoma. Int J Urol.

[23] Kim YB, Chang SK, Yang WI (2003). Primary NK/T cell lymphoma of the testis. A case report and review of the literature. Acta Haematol.

[24] Totonchi KF, Engel G, Weisenberg E (2002). Testicular natural killer/T-cell lymphoma, nasal type, of the natural killer-cell origin. Arch Pathol Lab Med.

[25] Guler G, Altinok G, Uner A (1999). CD56+ lymphoma presenting as a testicular tumor. Leuk Lymphoma.

[26] Chan JK, Tsang WY, Lau WH (1996). Aggressive T/natural killer cell lymphoma presenting as testicular tumor. Cancer.

[27] Lan SK, Lin CW, Ho HC (2008). Penile metastasis secondary to nasal NK/T-cell lymphoma. Urology.

[28] Chu L, Mao W, Vikramsingh KC (2013). Primary malignant lymphoma of glans penis: a rare case report and review of the literature. Asian J Androl.

[29] Gallardo F, Pujol RM, Barranco C (2009). Progressive painless swelling of glans penis uncommon clinical manifestation of systemic non Hodgkin’s lymphoma. Urology.

[30] Suzuki R, Takeuchi K, Ohshima K, Nakamura S (2008). Extranodal NK/T-cell lymphoma: diagnosis and treatment cues. Hematol Oncol.

[31] Mo ZY, Wang P, Yang HW (2016). Esophageal metastasis secondary to extranodal nasal-type natural killer/T-cell lymphoma: a case report. Mol Clin Oncol.

[32] Obama K, Tara M, Niina K (2003). L-asparaginase-based induction therapy for advanced extranodal NK/T-cell lymphoma. Int J Hematol.

[33] Fox CP, Boumendil A, Schmitz N (2015). High-dose therapy and autologous stem cell transplantation for extra-nodal NK/T lymphoma in patients from the western hemisphere: a study from the European Society for Blood and Marrow Transplantation. Leuk Lymphoma.

[34] Yhim HY, Kim JS, Mun YC (2015). Clinical outcomes and prognostic factors of up-front autologous stem cell transplantation in patients with extranodal natural killer/T cell lymphoma. Bio Blood Marrow Transplantation.

[35] Wang CB, Bai H, Xi R (2013). Curative efficacy for nasal type extranodal NK/T-cell lymphoma by autologous peripheral blood stem cell transplantation after sequencing chemotherapy and radiotherapy. Zhongguo Shi Yan Xue Ye Xue Za Zhi.

[36] Lee J, Au WY, Park MJ (2008). Autologous hematopoietic stem cell transplantation in extranodal natural killer/T cell lymphoma: a multinational, multicenter, matched controlled study. Biol Blood Marrow Transplant.

[37] Cui XZ, Wang HQ, Liu XM (2007). Treatment outcome and prognosis of autologous hematopoietic stem cell transplantation combined with high dose radiotherapy/chemotherapy in 22 patients with nasal NK/T cell lymphoma. Zhonghua Xue Ye Xue Za Zhi.

[38] Kim HJ, Bang SM, Lee J (2006). High-dose chemotherapy with autologous stem cell transplantation in extranodal NK/T-cell lymphoma: a retrospective comparison with non-transplantation cases. Bone Marrow Transplant.

